# Vitamin D Receptor Inhibits NLRP3 Activation by Impeding Its BRCC3-Mediated Deubiquitination

**DOI:** 10.3389/fimmu.2019.02783

**Published:** 2019-12-04

**Authors:** Zebing Rao, Xin Chen, Junxian Wu, Mengjun Xiao, Jing Zhang, Binghao Wang, Lei Fang, Hongjie Zhang, Xiaoming Wang, Shuo Yang, Yunzi Chen

**Affiliations:** ^1^Key Laboratory of Immune Microenvironment and Disease, Department of Immunology, Nanjing Medical University, Nanjing, China; ^2^Key Laboratory of Antibody Techniques of Ministry of Health, Nanjing Medical University, Nanjing, China; ^3^The State Key Laboratory of Pharmaceutical Biotechnology, School of Life Sciences, Nanjing University, Nanjing, China; ^4^Jiangsu Key Laboratory of Molecular Medicine, Medical School of Nanjing University, Nanjing, China; ^5^Department of Gastroenterology, The First Affiliated Hospital of Nanjing Medical University, Nanjing, China; ^6^Medical Centre for Digestive Diseases, Second Affiliated Hospital of Nanjing Medical University, Nanjing, China

**Keywords:** VDR, NLRP3 inflammasome, BRCC3, deubiquitinating, cytokines

## Abstract

The NLRP3 inflammasome is a multiprotein oligomer responsible for activation of the inflammatory response by promoting the maturation and secretion of the pro-inflammatory cytokines IL-1β and IL-18. Dysregulation of this inflammasome has been linked to several autoimmune diseases, indicating that NLRP3 is tightly regulated to prevent aberrant activation. The regulation of NLRP3 activation remains unclear. Here, we report the identification of vitamin D receptor (VDR) as a negative regulator of NLRP3 oligomerization and activation. VDR can physically bind NLRP3 and block the association of NLRP3 with BRCC3. When BRCC3-mediated deubiquitination of NLRP3 is inhibited by VDR, NLRP3 activation is subsequently inhibited. In the absence of VDR, caspase-1 activation and IL-1β release are increased in response to LPS-induced inflammation or alum-induced peritoneal inflammation, indicating that VDR is a negative regulator of NLRP3 inflammasome activation *in vivo*. In addition, vitamin D negatively regulates the NLRP3 inflammasome via VDR signaling to effectively inhibit IL-1β secretion. These studies demonstrate that VDR signaling constrains NLRP3 inflammasome activation and might be a potential treatment target for NLRP3 inflammasome-related diseases.

## Introduction

Pyroptosis is a highly inflammatory form of programmed cell death that promotes the rapid clearance of various bacterial and virus infections. The inflammasome is a multiprotein oligomer that serves as a platform for caspase-1-dependent activation of the maturation of the pro-inflammatory cytokines IL-1β and IL-18; the secretion of these cytokines results in pyroptosis (1). The NLRP3 inflammasome can be triggered by many different stimuli, such as amyloid-β, extracellular ATP, alum, nigericin (an antibiotic from *Streptomyces hygroscopicus*), and crystals (2, 3). Upon activation, NLRP3 assembles a typical multimeric inflammasome complex comprising the adaptor ASC and the effector pro-caspase-1; this complex mediates the proteolytic cleavage of pro-caspase-1 into active caspase-1 and converts the cytokine precursors pro-IL-1β and pro-IL-18 into biologically active IL-1β and IL-18 (4). Dysregulation of the inflammasome has been linked to several autoimmune diseases, such as types I and II diabetes, inflammatory bowel disease (IBD), gouty arthritis, multiple sclerosis, and vitiligo, as well as auto-inflammatory disorders (5–8). These diseases and disorders have been connected to the increased or decreased secretion of pro-inflammatory cytokines regulated by the inflammasome, indicating that NLRP3 inflammasome activation is tightly controlled in the normal state.

The regulation of NLRP3 inflammasome activation has been extensively investigated. Accumulating evidence indicates that the modification of NLRP3 occurs at the transcriptional and post-translational levels, with particular focus on ubiquitination, and phosphorylation (9). NLRP3 is poly-ubiquitinated with mixed Lys-48 and Lys-63 ubiquitin chains in resting macrophages (10, 11). A decrease in ubiquitinated NLRP3 can be induced by inflammasome activation signals (10). Inhibition of NLRP3 deubiquitination almost completely blocks NLRP3 activation in both mouse and human cells (10). BRCC3, a deubiquitinase, is crucial for NLRP3 activation at the post-transcriptional level due to its role in NLRP3 deubiquitination (10, 11). Although many studies have examined the regulation of NLRP3, the regulatory mechanism of NLRP3 activation remains unclear and requires further investigation.

To understand the signaling mechanism of NLRP3 inflammasome activation, we sought to identify proteins that interact with NLRP3. Using protein mass spectrometry analysis, we identified that NLRP3 can interact with vitamin D receptor (VDR). Beyond its well-established role in calcium–phosphorus homeostasis and bone metabolism, VDR plays anti-inflammatory roles in both innate and adaptive immunity (12, 13). VDR deficiency is associated with increased inflammation and deregulation in several inflammatory diseases, such as inflammatory bowel disease, sepsis, diabetes and asthma (14, 15). Vitamin D and VDR have anti-inflammatory effects and play an immunosuppressive role in autoimmunity. Together, they increase the phagocytic ability of monocytes to modulate the innate immune system (16, 17) and promote the ability of dendritic cells to modulate regulatory T cell differentiation (12, 14, 18, 19). Recent studies on vitamin D and VDR in inflammation-related diseases have received increasing attention.

Here, we demonstrate that VDR acts as an endogenous suppressor of NLRP3 inflammasome assembly to modulate NLRP3 activation. VDR directly interacts with NLRP3 and disturbs the association of NLRP3 with BRCC3, thereby inhibiting the deubiquitination of NLRP3 by BRCC3 and subsequently blocking activation of the NLRP3 inflammasome.

## Results

### VDR Interacts With NLRP3

**Figure 1:**
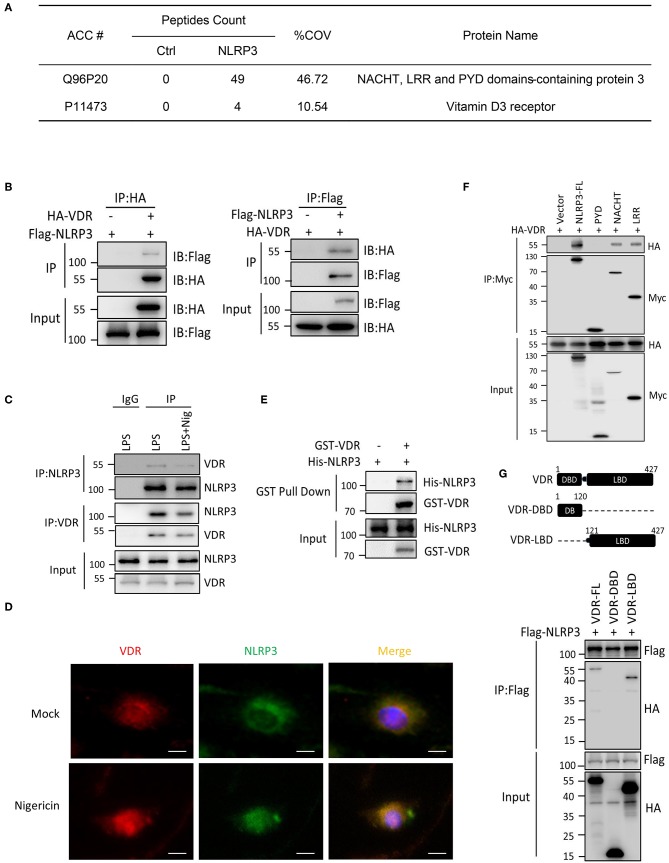
VDR interacts with NLRP3. **(A)** Mass spectrometry analysis of NLRP3 and VDR peptides after immunoprecipitation with Flag to pull down NLRP3-associated proteins in Flag-NLRP3-overexpressing HEK293T cells. **(B)** HA-VDR was co-expressed with Flag-tagged NLRP3 in HEK293T cells; proteins were immunoprecipitated and analyzed by immunoblotting. Whole-cell lysates are shown as the input. **(C)** LPS-primed BMDMs were unstimulated or stimulated with nigericin for 30 min. Cell lysates were immunoprecipitated (IP) and immunoblotted (IB) with the indicated antibodies. **(D)** Immunofluorescent staining for VDR and NLRP3 in LPS-primed BMDMs treated with or without nigericin. Scale bar, 10 μm. **(E)** Purified GST-VDR was incubated with purified His-NLRP3 for 2 h. His-NLRP3-Flag bound to GST-VDR was pulled down by glutathione beads and subjected to immunoblot analysis. **(F)** Wild-type or mutant NLRP3 (PYD, NACHT, or LRR) and HA-VDR were expressed in HEK293T cells, immunoprecipitated, and analyzed by immunoblotting. **(G)** Wild-type or mutant VDR (DBD or LBD) and Flag-NLRP3 were expressed in HEK293T cells, immunoprecipitated, and analyzed by immunoblotting.

To understand the signaling mechanism underlying NLRP3 inflammasome activation, we sought to identify proteins that interact with NLRP3. We expressed Flag-NLRP3 in HEK293T cells and performed Flag immunoprecipitation pull down NLRP3-associated proteins, which were evaluated using liquid chromatography–mass spectrometry. The analysis revealed VDR as a major interacting partner of NLRP3 (Figure 1A, Figure S1A). To investigate whether VDR binds other components of the NLRP3 complex, HA-VDR was co-transfected with NLRP3, ASC, or caspase-1 into HEK293T cells, and lysates were examined by co-immunoprecipitation. We found that overexpressed VDR interacted with NLRP3 (Figure 1B) but not with ASC or pro-caspase-1 (Figures S1B,C). The NLRP3–VDR interaction was also detected by endogenous immunoassay (Figure 1C), and the co-localization of these proteins in cells was observed in the immunofluorescence assay (Figure 1D, Figure S1E). To test whether the interaction is direct, we next performed a GST pull-down assay. NLRP3 was further confirmed to directly interact with VDR (Figure 1E). To identify the region of NLRP3 that associates with VDR, Myc-tagged wild-type, and mutant NLRP3 were co-expressed with HA-VDR in HEK293T cells, and immunoprecipitation experiments were performed. Interactions were observed between HA-VDR and full-length NLRP3 and NLRP3 mutated in the carboxy-terminal leucine-rich repeat (LRR) domain and nucleotide-binding domain (NACHT), while NLRP3 mutated in the amino-terminal pyrin domain (PYD) showed no interaction with HA-VDR (Figure 1F). On the other hand, the ligand binding domain (LBD), not the DNA binding domain (DBD), of VDR was required for the association with NLRP3 (Figure 1G). The PYD domain cannot bind to the LBD domain alone (Figure S1D). Our data indicated that VDR is a novel binding partner of NLRP3.

### VDR Inhibits NLRP3 Inflammasome-Mediated Caspase-1 Activation and IL-1β Secretion in Macrophages

**Figure 2:**
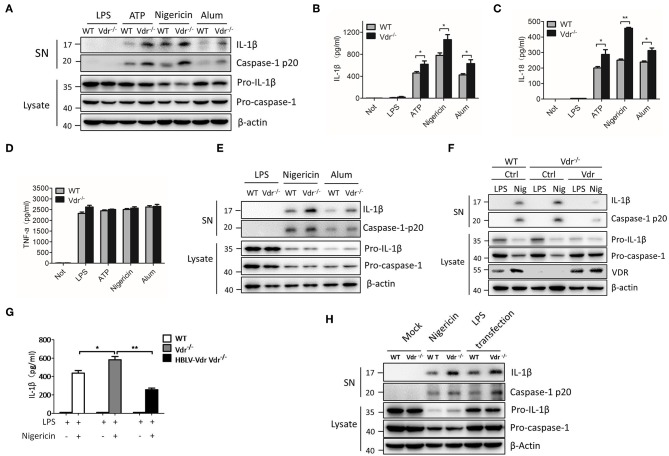
VDR inhibits NLRP3 inflammasome activation. **(A)** Immunoblot analysis of IL-1β and cleaved caspase-1 (p20) in culture supernatants (SN) of LPS-primed BMDMs (wild-type and Vdr^−/−^) treated for 4 h and then stimulated with ATP, nigericin, or alum. Immunoblot analysis of NLRP3, ASC, pro-IL-1β and pro-caspase-1 in cell lysates (Lysate). **(B–D)** IL-1β **(B)**, IL-18 **(C)**, and TNF-α **(D)** ELISAs using supernatants from LPS-primed BMDMs (wild-type and Vdr^−/−^) treated for 4 h and then stimulated with ATP, nigericin, or alum. **(E)** Immunoblot analysis of IL-1β and cleaved caspase-1 (p20) in culture supernatants (SN) of LPS-primed PMs (wild-type and Vdr^−/−^) treated for 4 h and then stimulated with nigericin or alum. Immunoblot analysis of pro-IL-1β and pro-caspase-1 in cell lysates (Lysate). **(F,G)** Immunoblot analysis of IL-1β and cleaved caspase-1 (p20) in culture supernatants (SN) of LPS-primed BMDMs **(F)**. Supernatants were analyzed by IL-1β ELISA **(G)**. **(H)** Pam3CSK4-primed BMDMs (wild-type and Vdr^−/−^) were stimulated by LPS transfection. Supernatants (SN) and cell extracts (Lysate) were analyzed by immunoblotting. Data are presented as the mean ± SEM; *p < 0.05, **p < 0.01, and ***p < 0.001. Data in **(B–D,G)** are representative of three independent experiments.

To examine the effect of VDR on inflammasome activation, we first checked the expression of components related to the NLRP3 complex in the absence of VDR. The results showed no significant differences in the proteins, including NLRP3, ASC, pro-IL-1β, and pro-caspase-1, in VDR-KO cells (Figure S2A). Next, BMDMs (bone marrow-derived macrophages) isolated from WT and Vdr^−/−^ mice were primed with LPS and then activated by NLRP3 stimuli, such as extracellular ATP, nigericin and alum, to evaluate NLRP3 activation. BMDMs from Vdr^−/−^ mice showed clear increases in caspase-1 cleavage and IL-1β (Figure 2A), and the secretion of IL-1β and IL-18 was significantly increased (Figures 2B,C). As a control for inflammasome-independent cytokines, TNF-α production was not affected (Figure 2D). Similar results were obtained in mouse peripheral macrophages (PMs) (Figure 2E). To confirm the ability of VDR to inhibit NLRP3 inflammasome activation, we restored VDR expression in VDR-KO BMDMs by lentivirus-mediated transduction. When NLRP3 inflammasomes were activated by nigericin, VDR overexpression decreased caspase-1 cleavage and IL-1β secretion (Figures 2F,G). Considering that non-canonical inflammasome activation is dependent on NLRP3 for IL-1β secretion, we induced non-canonical inflammasome activation with LPS in Pam3CSK4-primed BMDMs and found that IL-1β secretion was increased in Vdr^−/−^ BMDMs (Figure 2H). Taken together, these data suggested that the VDR inhibits NLRP3 inflammasome activation.

### VDR Blocks NLRP3-ASC Speck Formation

**Figure 3:**
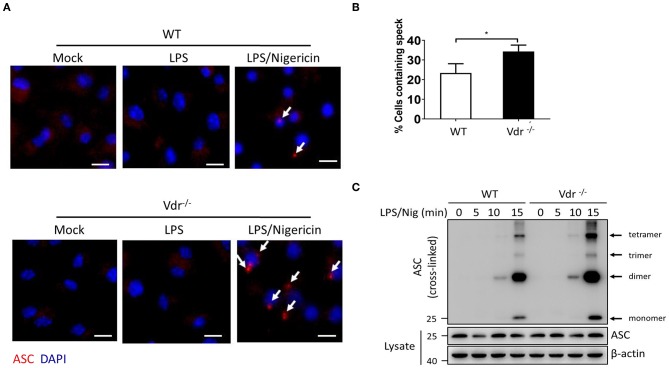
Vitamin D receptor blocks NLRP3 oligomerization and ASC speck formation. **(A,B)** Representative immunofluorescence images and quantification of endogenous ASC specks (arrows). The data show representative results from three combined independent experiments. Scale bar, 10 μm. **(C)** ASC oligomerization induced by the indicated stimuli at 0, 5, 10, and 15 min in WT and Vdr^−/−^ macrophages primed with LPS. Data are presented as the mean ± SEM; *p < 0.05. Data in panel B is representative of three independent experiments.

NLRP3 activators can induce the rapid formation of large intracellular ASC aggregates called ASC specks (20). In Vdr^−/−^ BMDMs, there was increased formation of ASC specks in the cytosol (Figures 3A,B). High-molecular-weight multiprotein complexes are assembled in activated inflammasomes (21), so we resolved cell lysates from WT and Vdr^−/−^ BMDMs by native polyacrylamide gel electrophoresis. In the stimulation time course experiment, more ASC oligomeric complexes were induced in Vdr^−/−^ BMDMs than in control BMDMs (Figure 3C), indicating that VDR is involved in the process of NLRP3 inflammasome assembly.

### VDR Interferes With the Association Between NLRP3 and BRCC3

**Figure 4:**
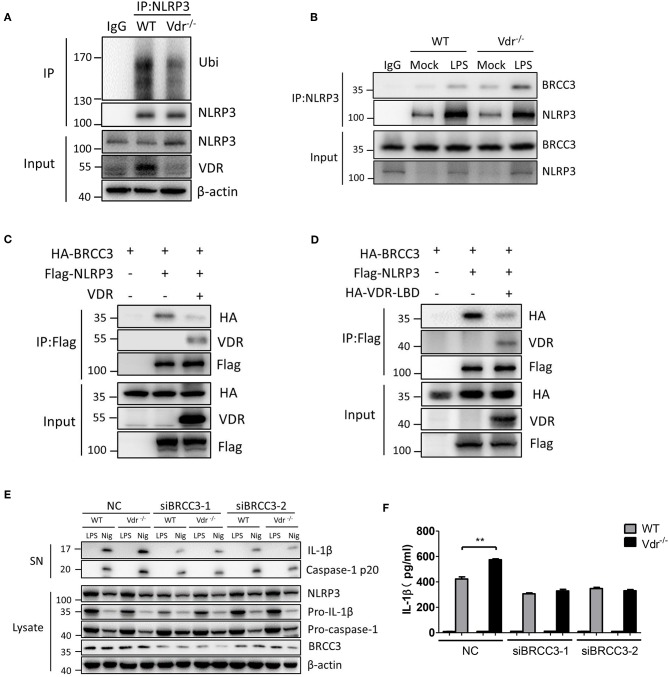
Vitamin D receptor interferes with the BRCC3–NLRP3 interaction. **(A)** Both WT and Vdr^−/−^ BMDMs were treated with LPS for 4 h. NLRP3 ubiquitination was analyzed. **(B)** Immunoblot analysis of BRCC3 protein in mock or LPS-primed WT and Vdr^−/−^ BMDMs lysates immunoprecipitated with the anti-NLRP3 antibody. **(C,D)** HEK293T cells were transfected with the indicated vectors. Samples were immunoprecipitated with the anti-Flag antibody and analyzed by immunoblotting. **(E)** LPS-primed BMDMs (wild-type and Vdr^−/−^) transfected with the indicated non-targeting or BRCC3-specific siRNA were unstimulated or stimulated with nigericin for 30 min. Supernatants (SN) and cell extracts (Lysate) were analyzed by immunoblotting. IL-1β ELISA **(F)**. Data are presented as the mean ± SEM; **p < 0.01. Data in **(F)** is representative of three independent experiments.

NLRP3 ubiquitination is a key inhibitor of NLRP3 inflammasome activation (10). In LPS-treated Vdr^−/−^ BMDMs, the ubiquitinated NLRP3 was decreased (Figure 4A), suggesting that VDR might be involved in the NLRP3 ubiquitination. Meanwhile, we found that VDR had no effect on the mRNA expressions of NLRP3-related deubiquitinase and ubiquitinase (Figures S3A–E), such as BRCC3, March7, Fbxl2, Trim31, and Pellino2 (22). BRCC3 is a deubiquitinating enzyme that critically deubiquitinates NLRP3 for NLRP3 inflammasome activation. To test whether VDR affects the association between NLRP3 and BRCC3, we analyzed this association in the presence of VDR. The results showed that VDR attenuated the binding of BRCC3 to NLRP3 (Figures 4B,C). Similarly, VDR-LBD also attenuated the interaction between BRCC3 and NLRP3, since this VDR domain was required for binding to NLRP3 (Figure 4D). To confirm the important role of the NLRP3–BRCC3 association in the VDR-mediated inhibition of NLRP3 inflammasome activation, we knocked down BRCC3 with siRNA and found that the increased caspase-1 cleavage and IL-1β secretion in Vdr^−/−^ BMDMs were eliminated (Figures 4E,F). NEK7 and PP2A interact with NLRP3 (23, 24). We found that VDR overexpression had no effect on the association of NEK7 or PP2A with NLRP3 (Figures S4A,B). Therefore, VDR affects the NLRP3 inflammasome by specifically blocking the association of NLRP3 with BRCC3. Therefore, we conclude that VDR interferes with the association between NLRP3 and BRCC3.

### VDR Inhibits NLRP3 Deubiquitination Mediated by BRCC3

**Figure 5:**
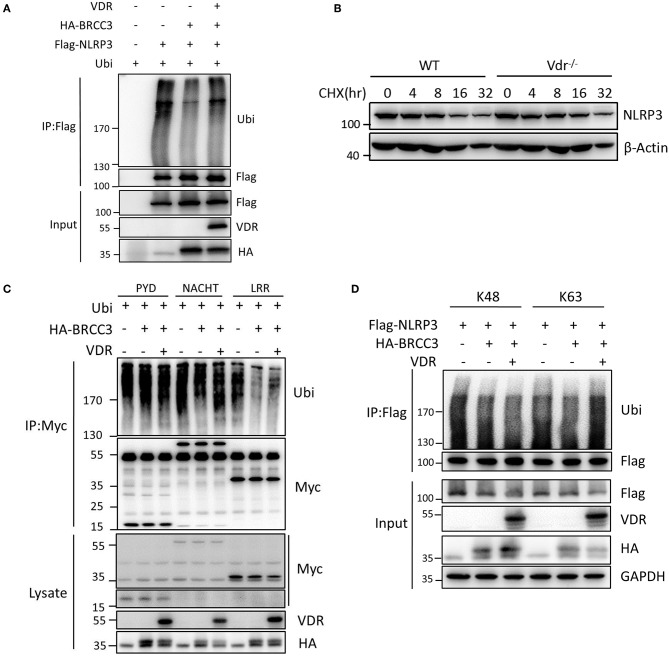
Vitamin D receptor inhibits NLRP3 deubiquitination. **(A)** HEK293T cells were transfected with the indicated vectors. NLRP3 ubiquitination was analyzed. **(B)** Immunoblot analysis of NLRP3 after treatment of LPS-primed BMDMs (wild-type and Vdr^−/−^) with CHX (1 μM) for the indicated time. **(C)** HEK293T cells expressing HA-BRCC3 and Myc-NLRP3 PYD, NACHT, or LRR were co-transfected with VDR as indicated. Myc-NLRP3 immunoprecipitants were analyzed for ubiquitination. **(D)** HEK293T cells expressing Flag-NLRP3, HA-BRCC3, and HA-Ubiquitin K63 or K48 were collected, and the cell lysates were immunoprecipitated with the anti-Flag antibody to detect ubiquitination.

To clarify that NLRP3 ubiquitination is regulated by VDR, we examined the effect of VDR on the BRCC3-mediated deubiquitination of NLRP3. Ubiquitin overexpression triggered the appearance of high apparent molecular weight NLRP3; however, the ubiquitination of Flag-NLRP3 was reduced upon BRCC3 addition (Figure 5A), which is consistent with the published report that BRCC3 promotes the deubiquitination of NLRP3. VDR overexpression recovered the level of NLRP3 ubiquitination, suggesting that the BRCC3-mediated deubiquitination of NLRP3 is inhibited by VDR (Figure 5A). We further examined the effects of VDR on the ubiquitination of different domains of NLRP3. Individual truncation mutants of NLRP3 (PYD, NACHT, or LRR) were overexpressed with ubiquitin in 293T cells. Only the ubiquitination of the LRR domain was downregulated by BRCC3 (Figure 5C); it was reported previously that BRCC3 mediates the deubiquitination of the LRR domain of NLRP3 (11). Consistently, VDR overexpression increased only LRR ubiquitination, not PYD or NACHT ubiquitination, demonstrating the key role of VDR in the BRCC3-mediated deubiquitination of NLRP3 (Figure 5C). In general, ubiquitination promotes protein degradation. Here, VDR deficiency had no effect on NLRP3 degradation in LPS-primed BMDMs (Figure 5B), suggesting that VDR-mediated inhibition of NLRP3 deubiquitination does not affect K48 ubiquitin chains. Consistent with this possibility, VDR overexpression markedly induced the modification of NLRP3 with K63 ubiquitin chains, not K48 ubiquitin chains (Figure 5D). Taken together, the data showed that VDR can inhibit BRCC3-mediated NLRP3 deubiquitination.

### VDR Deficiency Promotes NLRP3-Mediated Inflammation *in vivo*

**Figure 6:**
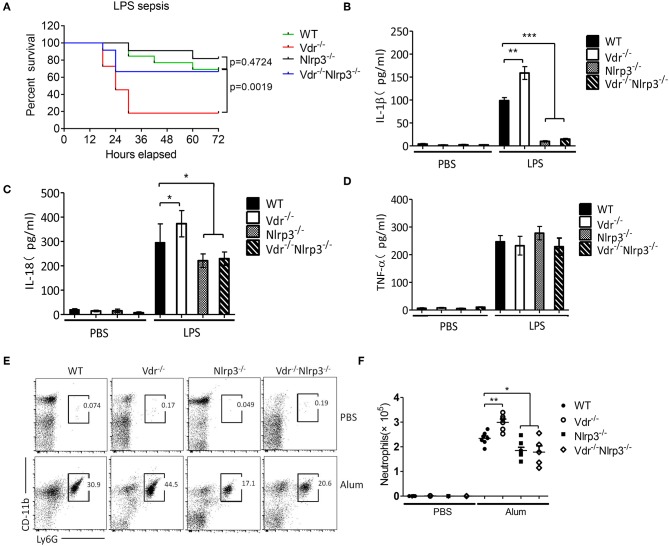
Vitamin D receptor deficiency promotes LPS-induced systemic inflammation and Alum-induced peritoneal inflammation via suppression of the NLRP3 inflammasome. **(A)** Survival of VDR^+/+^NLRP3^+/+^, VDR^−/−^NLRP3^+/+^, VDR^+/+^NLRP3^−/−^, and VDR^−/−^NLRP3^−/−^ mice (*n* = 10 mice/group) intraperitoneally injected with LPS (8 mg/kg body weight) over a period of 72 h. **(B–D)** Serum IL-1β, IL-18, and TNFα ELISAs at 6 h after intraperitoneal injection of LPS (8 mg/kg body weight) into VDR^+/+^NLRP3^+/+^, VDR^−/−^NLRP3^+/+^, VDR^+/+^NLRP3^−/−^, and VDR^−/−^NLRP3^−/−^ mice. **(E,F)** Representative FACS plots of peritoneal CD11b+ Ly6G+ cells (neutrophils) from VDR^+/+^NLRP3^+/+^, VDR^−/−^NLRP3^+/+^, VDR^+/+^NLRP3^−/−^, and VDR^−/−^NLRP3^−/−^ mice at 12 h after an intraperitoneal injection with alum (*n* = 5 mice/group). *p < 0.05, **p < 0.01, ***p < 0.001, NS *p* > 0.05. Values are the mean ± SEM of five mice per group. Both male and female mice were randomly assigned. Data in **(B–D,F)** are representative of three independent experiments.

To address the cross-talk between VDR and NLRP3 *in vivo*, we next induced sepsis in Vdr^−/−^, Nlrp3^−/−^, and Vdr^−/−^/Nlrp3^−/−^ mice by intraperitoneal injection of LPS (8 mg/kg). With this dose of LPS, there was no significant difference in survival between Nlrp3^−/−^ mice and WT. However, there was a marked reduction in survival in Vdr^−/−^ mice at 36 h (20%, compared to 90% in WT, *p*-value = 0.0019) that was partly rescued by Nlrp3 deficiency, as Vdr^−/−^/Nlrp3^−/−^ mice showed 70% survival at the same time point (Figure 6A). Our findings suggest that the role of VDR signaling in sepsis is largely dependent on NLRP3-induced inflammation. Moreover, we found that serum IL-1β and IL-18 levels were significantly increased in Vdr^−/−^ mice but not in NLRP3^−/−^ or Vdr^−/−^NLRP3^−/−^ mice (Figures 6B,C). As a control, serum TNF-α production showed no significant difference among groups (Figure 6D). We next addressed the negative role of VDR in NLRP3 inflammasome activation using an alum-induced peritonitis model. Peritoneal neutrophils in lavage samples are an indicator of NLRP3-induced inflammation in response to the intraperitoneal injection of alum, so we examined the recruitment of peritoneal neutrophils. CD11b^+^ Ly6G^+^ cells (neutrophils) were increased in Vdr^−/−^ mice but decreased in NLRP3^−/−^ and Vdr^−/−^NLRP3^−/−^ mice compared to WT mice (Figures 6E,F). These results demonstrated that VDR inhibits NLRP3-induced inflammation *in vivo*.

### Vitamin D Enhances the VDR-Mediated Inhibition of NLRP3 Inflammasome Activation

**Figure 7:**
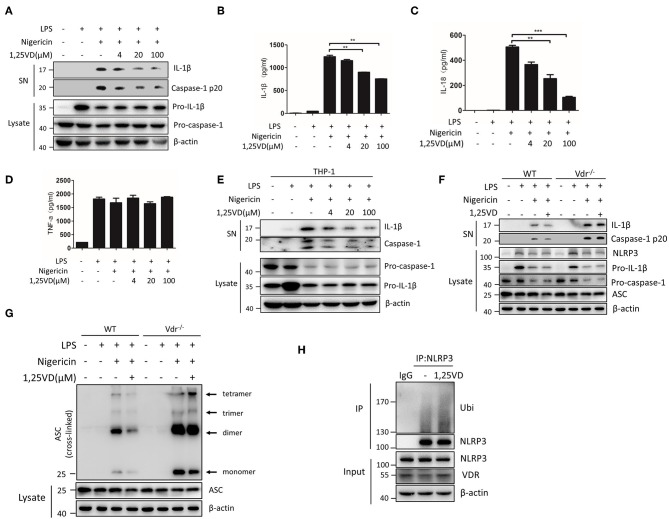
Vitamin D enhances VDR-mediated inhibition of NLRP3 inflammasome activation. **(A)** Immunoblot analysis of IL-1β and cleaved caspase-1 (p20) in culture supernatants (SN) of LPS-primed BMDMs treated for 3 h with various doses (upper lanes) of 1,25 VD and then stimulated with nigericin. Immunoblot analysis of NLRP3, ASC, pro-IL-1β and pro-caspase-1 in cell lysates (Lysate). **(B–D)** IL-1β **(B)**, IL-18 **(C)**, and TNF-α **(D)** in supernatants. **(E)** LPS-primed BMDMs (wild type and Vdr^−/−^) were treated with 1,25 vitamin D (1,25 VD, 20 μM) and then stimulated with nigericin. Supernatants (SN) and cell extracts (Lysate) were analyzed by immunoblotting. **(F)** LPS-primed THP-1 cells were treated with different doses of 1,25 vitamin D (1,25VD) as indicated and then stimulated with nigericin. Supernatants (SN) and cell extracts (Lysate) were analyzed by immunoblotting. **(G)** The induction of ASC oligomerization by the indicated stimuli in WT and Vdr^−/−^ LPS-primed macrophages. Nigericin stimulation lasted 15 min. **(H)** LPS-primed BMDMs were treated with or without 20 μM 1,25VD for 3 h. NLRP3 ubiquitination was analyzed. Data are presented as the mean ± SEM; *p < 0.05, **p < 0.01, and ***p < 0.001. Data in **(B–D)** are representative of three independent experiments.

Vitamin D is a VDR ligand, and its active metabolite is 1,25(OH)2D3. In general, vitamin D binds to VDR to activate VDR signaling. When BMDMs were treated with 1,25(OH)2D3, NLRP3 inflammasome activation by LPS and nigericin was gradually inhibited, and IL-1β and caspase-1 cleavage decreased in a dose-dependent manner (Figures 7A–C). TNF-α production was not affected by 1,25(OH)2D3 (Figure 7D). In human THP-1-derived macrophages, we reconfirmed that vitamin D significantly attenuated NLRP3 inflammasome activation (Figure 7E). However, 1,25(OH)2D3 treatment had no effect on NLRP3 inflammasome activation or ASC oligomeric complex formation in Vdr^−/−^ BMDMs (Figures 7F,G), suggesting that vitamin D is dependent on VDR to regulate NLRP3 inflammasome activation. Furthermore, 1,25(OH)2D3 dramatically inhibited ASC oligomeric complex formation (Figure 7G) and increased the ubiquitination of NLRP3 in LPS-primed BMDMs (Figure 7H). These results suggest that 1,25(OH)2D3 enhances the VDR-mediated inhibition of NLRP3 inflammasome activation.

## Discussion

The NLRP3 inflammasome is activated by numerous PAMPs and DAMPs and plays a key role in host defense (25–28). NLRP3 inflammasome activation can be regulated at the post-translational and transcriptional levels by several molecules, such as bile acids, dopamine, nitric oxide, type I IFNs, SHP, and A20 (29–34). Here, we report a novel role for VDR as a negative regulator of NLRP3 inflammasome activation in macrophages. Our data demonstrate that VDR directly interacts with NLRP3, inhibits NLRP3 inflammasome assembly, and decreases the NLRP3-mediated secretion of IL-1β and IL-18, suggesting that VDR might be a potential anti-inflammatory factor.

Recently, a two-step model of NLRP3 activation has been well documented, which is triggered by two sequential signals. The first step, NLRP3 needs to be “primed” by Toll-like receptor (TLR) agonists such as LPS. Activation of TLR signaling not only transcriptionally upregulates NLRP3 expression, but also post-transcriptionally activates NLRP3 by phosphorylation and deubiquitination. The second step, defined as “activation,” can be induced by several potent stimuli such as nigericin, leading to the oligomerization of NLRP3, and the subsequent assembly of inflammasome (35, 36). The post-translational modification of NLRP3 is essential for inflammasome assembly and activation. Studies demonstrate that NLRP3 is both K48 and K63 ubiquitinated, suggesting that regulation may be more complex (11, 30, 37, 38). Increasing evidence has shown that BRCC3-mediated NLRP3 deubiquitination is critical for inflammasome activation (10, 11). In this study, we demonstrate that BRCC3–NLRP3 complex formation is interrupted by VDR via competition with BRCC3 for NLRP3 binding. The association of NEK or PP2A with NLRP3 is not affected by VDR, so VDR has a special role in regulating BRCC3-mediated NLRP3 activation. BRCC3 promotes inflammasome activation by deubiquitinating NLRP3. Notably, the decreases in IL-1β secretion and NLRP3 ubiquitination induced by VDR deficiency were almost completely eliminated in BRCC3 knockdown cells, which suggests that VDR-mediated inhibition of NLRP3 activation is mainly due to disruption of BRCC3–NLRP3 complex formation. Thus, our findings provide a novel mechanism of regulating NLRP3 activation by controlling BRCC3-mediated deubiquitination.

Notably, several nuclear receptors have been reported to selectively or cooperatively regulate NLRP3 inflammasome activation, including transmembrane G protein coupled receptor-5 (TGR5), farnesoid X receptor (FXR), and the orphan nuclear receptor SHP (30, 32, 39). SHP inhibits NLRP3 inflammasome assembly and NLRP3-dependent IL-1β maturation in the presence of a variety of inflammasome-activating stimuli. TGR5 signaling prevents metabolic disorder by inhibiting NLRP3 inflammasome in a manner dependent on cAMP-PKA. Consistently, VDR is another nuclear receptor that has been shown to be a post-translational regulator of NLRP3 inflammatory responses. Collectively, all these nuclear receptors function as an adaptor associated with the NLRP3 complex. These data suggest the possibility that nuclear receptors might respond to cellular stress in a transcription-independent manner. VDR, an endogenous regulator of NLRP3, mitigates inflammasome-related inflammation *in vivo*, such as LPS-induced systemic inflammation and alum-induced peritoneal inflammation.

Vitamin D initiates biological responses by binding to VDR (40). In this study, we also found that vitamin D blocked NLRP3 deubiquitination and activation dependent on VDR (Figure 7F). Vitamin D/VDR system enhances intestinal transcellular transport of calcium and high levels of calcium stimulate inflammasome activation (41, 42). But VDR-deficient mice are hypocalcemic, and the inflammasome activation is increased in VDR-deficient mice, suggesting that VDR inhibits the inflammasome activation in a calcium-independent manner. Collectively, we demonstrate that vitamin D can inhibit NLRP3 inflammasome activation via VDR signaling, suggesting that vitamin D treatment is a possible strategy for the treatment of NLRP3-related inflammatory diseases.

## Methods

### Mice

*Vdr*^−/−^ were obtained from The Jackson Laboratory. *Nlrp3*^−/−i^ mice were a kind gift from Dr. Vishva M. Dixit (Genentech). Mice were housed in a specific pathogen-free environment in the Animal Core Facility of Nanjing Medical University. All animal experiments were approved by the Institutional Animal Care and Use Committee of Nanjing Medical University. Mice were used at 6–8 weeks of age.

### Reagents

LPS (O111:B4, L2630), ATP (A7699), disuccinimidyl suberate (S1885), nigericin (N7143), and phorbol myristate acetate (PMA, P1585) were from Sigma-Aldrich; alum (77161) and the c-Myc tag (MA1-980) were from Thermo Fisher Scientific; and poly(dA:dT) was from InvivoGen. The following antibodies were used: anti-Flag (F1804) (Sigma); anti-mouse caspase-1 p20 (AG-20B-0042) and anti-mouse NLRP3 (AG-20B-0014) (AdipoGen); anti-human caspase-1 (ab108362) and anti-BRCC36 (ab108411) (Abcam); anti-mouse IL-1β (AF-401-NA) (R&D); anti-HA-tag (3724) (Cell Signaling Technology); and anti-VDR (sc-13133), anti-ASC (sc-514414), and anti-β-actin (sc-47778) (Santa Cruz). The Mouse TNF ELISA Kit (558534), Mouse IL-1β ELISA Kit (559603), and TMB Substrate Reagent Set (555214) were from BD Biosciences. The mouse IL-18 ELISA set (EMC06) was from ExCell Biotech (Table 1).

**Table 1:** Reagents.

**Reagent or resource**	**Source**	**Identifier**
**Antibodies**		
IL-1β	R&D Systems	Cat# AF-401-NA
Caspase-1(mouse)	Adipogen	Cat# AG-20B-0044
NLRP3	Adipogen	Cat# AG-20B-0014
Caspase-1(human)	Abcam	Cat# ab108362
BRCC36	Abcam	Cat# ab108411
HA	Cell Signaling Technology	Cat#3724
VDR	Santa Cruz	Cat# sc-13133
ASC	Santa Cruz	Cat# sc-514414
β-actin	Santa Cruz	Cat# sc-47778
Flag	Sigma	Cat# F1804
c-Myc	Thermo Fisher Scientific	Cat# MA1-980
**Chemicals, peptides, and recombinant proteins**		
ATP	Sigma	Cat# A7699
LPS	Sigma	Cat# L2630
Alum	Thermo Fisher Scientific	Cat#77161
Nigericin	Sigma	Cat# N7143
PMA	Sigma	Cat# P1585
Pam3CSK4	Invivogen	
**Critical Commercial Assays**		
TNF ELISA kit	BD Biosciences	Cat#558534
Mouse IL-1β ELISA kit	BD Biosciences	Cat#559603
TMB Substrate Reagent Set	BD Biosciences	Cat#555214
Mouse IL-18 ELISA kit	ExCell Biotech	EMC06
**Experimental models: cell lines**		
HEK293T cells	ATCC	CRL-11268
THP1 cells	ATCC	TIB-202
**Experimental models: organisms/strains**		
Mouse: Nlrp3-/-	Dr. Vishva M. Dixit labs(Genetech)	N/A
Mouse: Vdr-/-	Jackson Laboratory	JAX:017969
**Oligonucleotides**		
MouseBRCC3siRNA1(sense) (Sequence: GGCAGAAAGGUUGGCUGAATT)	This paper	N/A
MouseBRCC3siRNA1(antisense) (Sequence: UUCAGCCAACCUUUCUGCCTT)	This paper	N/A
MouseBRCC3siRNA2(sense) (Sequence: GGAAGAACAGGAUGCAUAUTT)	This paper	N/A
MouseBRCC3siRNA2(sense) (Sequence: AUAUGCAUCCUGUUCUUCCTT)	This paper	N/A
**Recombinant DNA**		
PCMV-HA-VDR	(44)	N/A
Flag-NLRP3	Professor Paul N. Moynagh (National University of Ireland Maynooth, Ireland)	N/A
HA-ASC	Professor Paul N. Moynagh (National University of Ireland Maynooth, Ireland)	N/A
HA-ubiquitin	Professor Paul N. Moynagh (National University of Ireland Maynooth, Ireland)	N/A
Myc-pp2a	Professor Paul N. Moynagh (National University of Ireland Maynooth, Ireland)	N/A
pCMV-HA-BRCC3	This paper	N/A
pCMV-HA-NEK7	This paper	N/A
Pgex6p-1-GST-VDR	This paper	N/A
Pet28a-His-NLRP3	This paper	N/A

### Cell Culture

Primary BMDMs were generated as described previously (43). The mouse tibia and femur were isolated and flushed with cold PBS through a 25-G needle in a sterile environment. Cells were cultured in DMEM supplemented with 10% FBS, 1% penicillin/streptomycin, and 10% (v/v) conditioned medium from L929 mouse fibroblasts for 6 days. The medium was replaced every 2 days. Peritoneal macrophages were harvested by injecting 5 ml of sterile PBS into the peritoneal cavity. Then, the cells were centrifuged at 1,000×g for 5 min, resuspended in RPMI containing 10% (v/v) fetal bovine serum, and cultured at 37°C. For the inflammasome activation assay, 1 × 10^6^ cells were plated in 12-well plates overnight. Then, the cells were primed with LPS (200 ng/ml) for 4 h and then stimulated with PBS (mock), 10 mM ATP (30 min), 10 μM nigericin (1 h), alum (250 μg/ml, 6 h). For non-canonical inflammasome activation, cells were primed with 100 ng/ml Pam3CSK4 (InvivoGen) for 4 h, after which 2 μg/ml LPS was added with Lipofectamine 2000 (Invitrogen) for 16 h (30). Cell lysates and supernatants were analyzed by Western blot analysis.

### Plasmid Construction and Transfection

The PCMV-HA-VDR plasmid was reported previously (44). Flag-NLRP3, HA-ASC, HA-ubiquitin, and Myc-pp2a were provided by Professor Paul N. Moynagh (National University of Ireland, Maynooth, Ireland). Recombinant vectors encoding human NEK7 and human BRCC3 were constructed by PCR-based amplification of complementary DNA from THP-1 cells and then cloned into the pCMV-HA eukaryotic expression vector. pGEX6p-1-GST-VDR, pET28a-His-NLRP3, and truncated mutants of NLRP3 and VDR were generated by the Original TA Cloning Kit (Vazyme Biotech). After plasmid construction, the mutation in the gene encoding VDR was introduced into each expression vector by the Fast Mutagenesis Kit V2 (Vazyme Biotech). All plasmid constructs were confirmed by DNA sequencing. Plasmids were transiently transfected into HEK293T cells with PolyJet reagents (SignaGen) according to the manufacturer's instructions.

### Mass Spectrometry Analysis of NLRP3-Associated Proteins

Empty Flag constructs or Flag-NLRP3 were transfected into HEK293T cells for 24 h, and the cells were collected and resuspended in lysis buffer [50 mM Tris–HCl (pH 6.8), 2% (w/v) SDS, 0.1% (w/v) bromophenol blue, 10% (v/v) glycerol, and 100 mM DTT (dithiothreitol)]. Extracts were immunoprecipitated with anti-Flag antibody and Protein A/G-Agarose beads and then dissolved in sample buffer. IP-enriched protein complexes were separated by SDS-PAGE, visualized by Coomassie blue staining, and then excised for in-gel digestion with trypsin. The peptides were extracted from gel bands and subjected to LC-MS/MS analysis. Tryptic peptides were separated on a C18 column and analyzed by an LTQ Orbitrap Velos mass spectrometer (Thermo). The resulting MS/MS data were processed using ProteinPilot^TM^ software 4.5 (AB Sciex). The original MS/MS data were submitted to ProteinPilot (version 4.5, AB Sciex) for data analysis and searched against *Homo sapiens* in the UniProt database (http://www.uniprot.org/proteomes/UP000005640).

### ELISA

The concentrations of mouse IL-1β and mouse TNF-α were measured using ELISA kits (BD Systems), and the concentration of mouse IL-18 was measured using ExCell Biotech kits according to the manufacturer's instructions.

### ASC Oligomerization Assay

Cells were plated on 12-well plates and stimulated as indicated. Cells were washed three times with PBS and lysed in PBS containing 0.5% Triton X-100 for 30 min at 4°C. The cell lysates were centrifuged at 8000×*g* for 15 min at 4°C (24). Triton X-100-insoluble pellets were washed twice with PBS and suspended in 200 μl of PBS. The pellets were then cross-linked at room temperature for 30 min by adding fresh disuccinimidyl suberate (2 mM). The cross-linked pellets were centrifuged at 8000×*g* for 15 min and lysed in 2× sample buffer for Western blot analysis of ASC oligomers.

### RNA Quantitation

Total RNA was extracted from BMDMs using TRIzol reagent (Life Technologies). cDNA was generated from total extracted RNA using HiScript II Q RT SuperMix for qPCR (Vazyme Biotech Co., Ltd) according to the manufacturer's protocol. Quantitative PCR was performed with ChamQ SYBR qPCR Master Mix (Vazyme Biotech Co., Ltd) using a Real-Time PCR System (StepOne, Applied Biosystems). The sequences of the PCR primers are listed in Table 2. Data were normalized to β-actin expression in each sample.

**Table 2:** Primers.

Nlrp3	Forward 5′-TGGATGGGTTTGCTGGGAT-3′ Reverse 5′-CTGCGTGTAGCGACTGTTGAG-3′
Trim31	Forward 5′-CCAGAGTCAAACCGTGAGCG−3′ Reverse 5′- GGCAACTTGGAGCCCGAA-3′
March7	Forward 5′-GACAGTACCAAGTTCTAGGGACT-3′ Reverse 5′-AGTTGTACGCCTACCTTCATTG-3′
Fbxl2	Forward 5′-CAGTGATGATGGCCTTATCAACA-3′ Reverse 5′-TGGAAGTTAAAAAGATCCACCCG-3′
Pellino2	Forward 5′-ACCAACGGTGTCCTGGTGATG-3′ Reverse 5′-CCTGGTCTCTCGCAAGGTGTA-3′
Brcc3	Forward 5′-GTGCAGGCGGTTCATCTTGA-3′ Reverse 5′-AACTCCCCTATACACAGACCC-3′
β-actin	Forward 5′-TGTTACCAACTGGGACGACA-3′ Reverse 5′-CTGGGTCATCTTTTCACGGT-3′

### Immunoprecipitation and Immunoblot Analysis

For whole-cell lysate analysis, cells were lysed in SDS lysis buffer [50 mM Tris–HCl (pH 6.8), 2% (w/v) SDS, 0.1% (w/v) bromophenol blue, 10% (v/v) glycerol, and 100 mM DTT]. For co-immunoprecipitation, cells were treated as indicated and then collected in 500 μl of RIPA Lysis Buffer [50 mM Tris (pH 7.4), 150 mM NaCl, 1% NP-40, and 0.25% sodium deoxycholate], followed by incubation at 4°C for 30 min. After centrifugation for 12 min at 12,000×*g*, the supernatants were collected and incubated with the appropriate antibody overnight. The immunocomplexes were captured by the addition of 30 μl of Protein A/G-Agarose slurry and gentle rotation for 90 min at 4°C. The agarose beads were collected by centrifugation for 3 min at 100 rpm. The supernatant was discarded, and the beads were washed three times with 800 μl of ice-cold RIPA buffer and twice with PBS. The agarose beads were resuspended in 50 μl of SDS loading buffer and mixed gently. The samples were separated by SDS-PAGE, transferred to polyvinylidene difluoride (PVDF) membranes (Millipore), and analyzed by immunoblot. Immunoreactivity was visualized by the Tanon Imaging System. For the NLRP3 ubiquitination assay, cells were collected in 300 μl of RIPA buffer. After centrifugation for 10 min, the supernatants were treated with 1% (w/v) SDS, heated to 95°C for 5 min to dissociate NLRP3 from any associated proteins, and then diluted 10-fold in RIPA buffer before immunoprecipitation.

### Immunofluorescence Staining and Confocal Analysis

For ASC speck analysis, BMDMs were plated on coverslips. The cells were fixed for 20 min with 4% paraformaldehyde and then permeabilized with 0.2% NP-40/PBS for 10 min. The cells were incubated with anti-rabbit ASC antibody (1:200) overnight, followed by incubation with anti-rabbit Cy3-conjugated AffiniPure (Jackson ImmunoResearch). Nuclei were stained with DAPI (4′,6′-diamidino-2-phenylindole hydrochloride; Sigma-Aldrich). HEK293T cells transiently transfected with plasmids encoding Flag-NLRP3, HA-ASC, and GFP-VDR were cultured for 24 h. The cells were incubated overnight with anti-Flag and anti-HA antibodies (1:100) and then incubated with fluorescent secondary antibody. The cells were examined with confocal laser microscopy (LSM710, Carl Zeiss, Germany).

### GST Pull-Down Assay

sBL21(DE3) cells were transformed with pGEX-6P1 bacterial expression plasmids encoding GST or GST-human VDR. Recombinant protein expression was induced by 0.1 mM IPTG for 8 h at 20°C. Human NLRP3 was cloned into pET28a bacterial expression plasmids. Recombinant protein expression was induced by 0.1 mM IPTG for 8 h at 26°C. Bacteria were pelleted and lysed with PBS containing 1 mM PMSF, 1 mM DTT, and 1% Triton X-100. Recombinant His-NLRP3 protein was incubated on a rotator at 4°C overnight with GST and GST-VDR. GST proteins were pulled down using Glutathione MagBeads (GenScript). Beads were washed three times with pull-down buffer and twice with PBS before being analyzed by immunoblot.

### Retroviral Rescue Assay

Murine Vdr was sub-cloned into the pHBLV-CMV-MCS-3XFlag-GFP-PURO lentivirus vector. Lentivirus-containing medium was obtained from Hanbio. On the first day, WT and Vdr-deficient BMDMs were plated in 12-well plates. On the second day, lentivirus containing the Vdr construct or empty vector was incubated with BMDMs (MOI = 50) at 37°C for 4 h. After 4 h, the medium was replaced with fresh medium, and the cells were incubated for another 48 h. The lysates were analyzed by immunoblot.

### siRNA

siRNAs were ordered from GenePharma. The siRNA sequences were as follows: BRCC3-1 (sense), GGCAGAAAGGUUGGCUGAATT; BRCC3-1 (antisense), UUCAGCCAACCUUUCUGCCTT; BRCC3-2 (sense), GGAAGAACAGGAUGCAUAUTT; BRCC3-2 (antisense), AUAUGCAUCCUGUUCUUCCTT; Stealth RNAi negative control (sense), UUCUCCGAACGUGUCACGUTT; and negative control (antisense), ACGUGACACGUUCGGAGAATT. BMDMs were plated in 12-well plates and transfected with 100 nM siRNA using 5 μl of Lipofectamine RNAiMAX (Invitrogen) in OPTI-MEM. The medium was changed 6 h later, and the cells were cultured for 48 h in RPMI1640 medium supplemented with 10% FBS and 10% L929 medium.

### *In vivo* LPS Challenge

For the survival analysis, C57BL/6J mice aged 6–8 weeks were administered 8 mg/kg LPS by intraperitoneal injection and monitored for 72 h. The moribund state, defined by (1) hypothermia, (2) inability to roll over from side to chest, or (3) dyspnea/labored breathing, was used as humane endpoint. For cytokine analysis, serum samples were collected after 6 h, and cytokines were measured by ELISA.

### Intraperitoneal Leukocyte Recruitment

C57BL/6J mice aged 6–8 weeks were intraperitoneally injected with 700 mg of alum and evaluated 12 h later. The peritoneal cavity was washed with 5 ml of PBS. Peritoneal exudate cells (PECs) were analyzed by flow cytometry. The recruitment of neutrophils was visualized with CD11b-PE (M1/70; BD Biosciences) and Ly-6G-APC (1A8; eBioscience). PI (Sigma) was used to exclude dead cells. Neutrophil (CD11b^+^Ly6G^+^) recruitment was analyzed on a CytoFLEX (Beckman Coulter) (45).

### Ethics Statement

This study was carried out in accordance with the recommendations of the guidelines of the Animal Care Committee of Nanjing Medical University, Jiangsu, China.

### Statistical Analyses

The results are expressed as the mean ± SEM. Statistical analyses were carried out using Student's *t*-test. Data were considered significant at *p* ≤ 0.05 (*), *p* ≤ 0.01 (**), and *p* ≤ 0.001 (***).

## Data Availability Statement

The raw data supporting the conclusions of this article will be made available by the authors, without undue reservation, to any qualified researcher.

## Ethics Statement

The animal study was reviewed and approved by the Animal Care Committee of Nanjing Medical University.

## Author Contributions

YC and ZR designed the research, analyzed data, and wrote the paper. XC, JW, MX, JZ, BW, and LF provided research reagents and technical assistance. HZ, XW, and SY assisted in data analysis and manuscript preparation. YC was responsible for the overall research design, data analysis, and paper preparation.

### Conflict of Interest

The authors declare that the research was conducted in the absence of any commercial or financial relationships that could be construed as a potential conflict of interest.
