# Supplementary material for: Vitamin D Receptor Inhibits NLRP3 Activation by Impeding Its BRCC3-Mediated Deubiquitination
Source: Front Immunol. 2019 Dec 4;10:2783. doi: 10.3389/fimmu.2019.02783 (PMC6904361; doi:10.3389/fimmu.2019.02783)
Supplement: Supplementary file 1 [file Data_Sheet_1.pdf]

Vitamin D receptor inhibits NLRP3 activation by impeding Its BRCC3-mediated deubiquitination

Zebing Rao1,2,Xin Chen1,2,Junxian Wu1,2,Mengjun Xiao1,2,Jing Zhang4,Binghao Wang6,,Lei Fang6,Hongjie Zhang5Xiaoming Wang1,2,Shuo Yang1,2*and Yunzi Chen1,2,3*

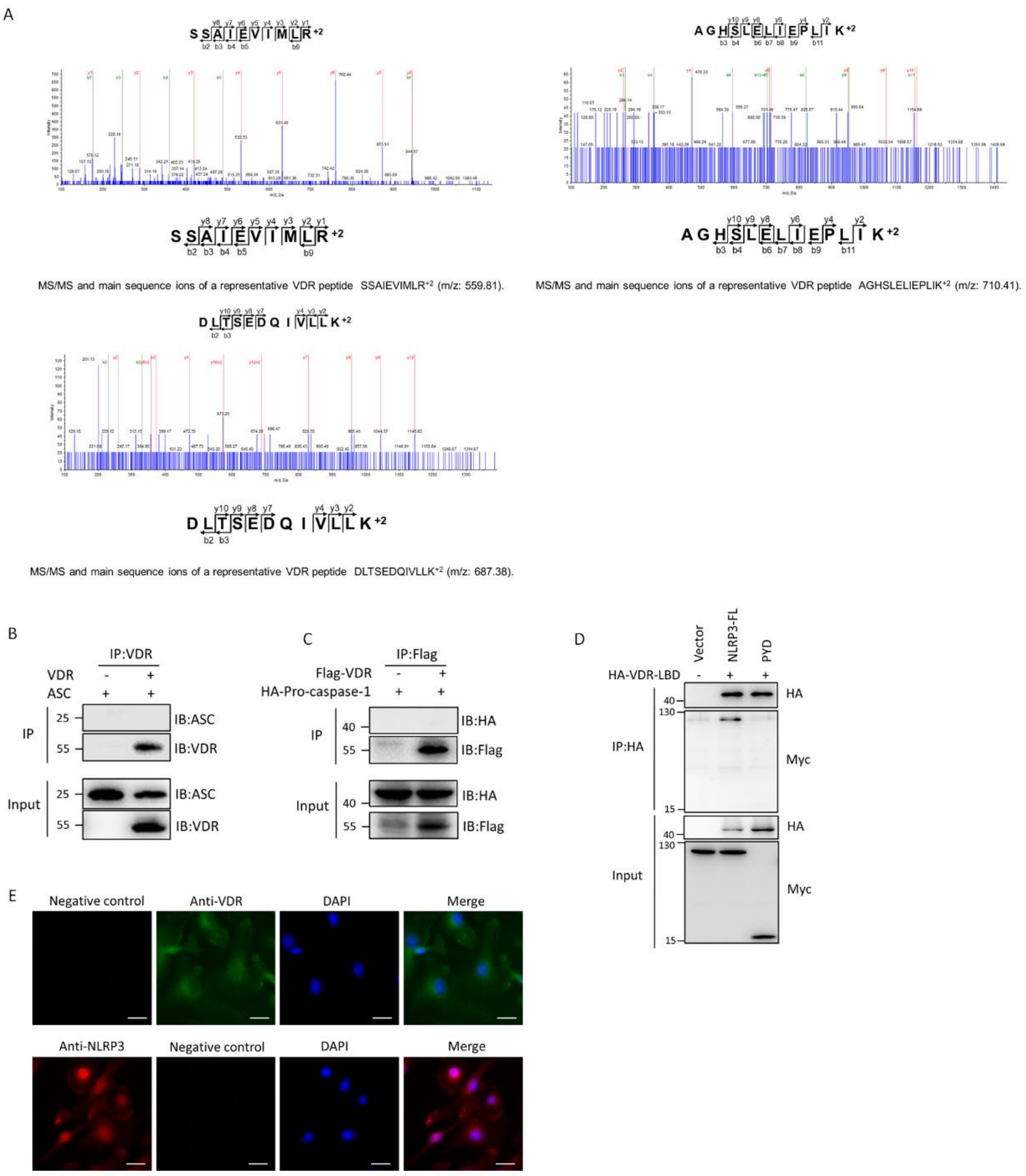

FIGURE S1. VDR does not interact with ASC or pro-caspase-1, related to Figure 1

(A) Mass spectrometry analysis of NLRP3 with Flag to pull down NLRP3-associated

proteins in Flag-NLRP3-overexpressing HEK293T cells. MS/MS map and main sequence ions of a representative VDR peptide.

(B) Flag-VDR was co-expressed with HA-tagged ASC in HEK293T cells, and proteins were immunoprecipitated and analysed by immunoblotting. Whole-cell lysates are shown as the input.

(C) Flag-VDR was co-expressed with HA-tagged pro-caspase-1 in HEK293T cells, and proteins were immunoprecipitated and analysed by immunoblotting. Whole-cell lysates are shown as the input.

(D) Wild-type or mutant NLRP3 (PYD) and HA-VDR-LBD were expressed in HEK293T cells, immunoprecipitated and analysed by immunoblotting.

(E) Negative control for anti-VDR and anti-NLRP3 in Immunofluorescence with isotype antibody. The cells were stained with isotype antibody and second antibody, the nuclei were visualized with DAPI staining. Scale bar, 20 μm.

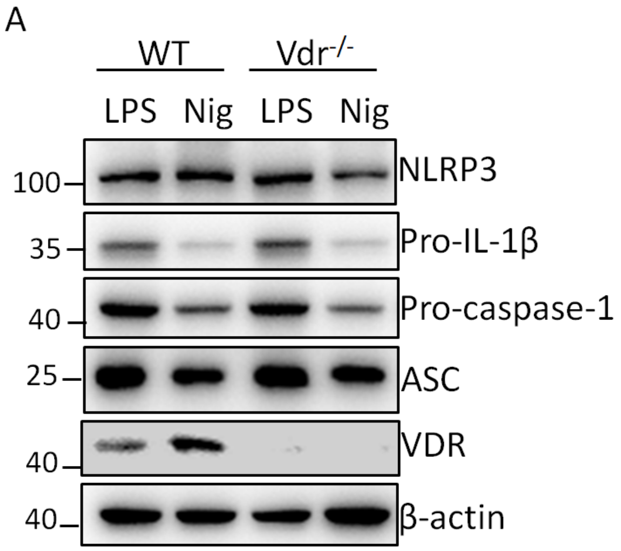

FIGURE S2. NLRP3, ASC, pro-IL-1β and pro-caspase-1 protein expression in BMDMs (wild-type and Vdr$^{-/-}$): Related to Figure 2

(A) LPS-primed BMDMs (wild-type and Vdr$^{-/-}$) were stimulated with nigericin. The protein levels of NLRP3, ASC, pro-IL-1β and pro-caspase-1 in whole-cell lysates were analysed by immunoblotting.

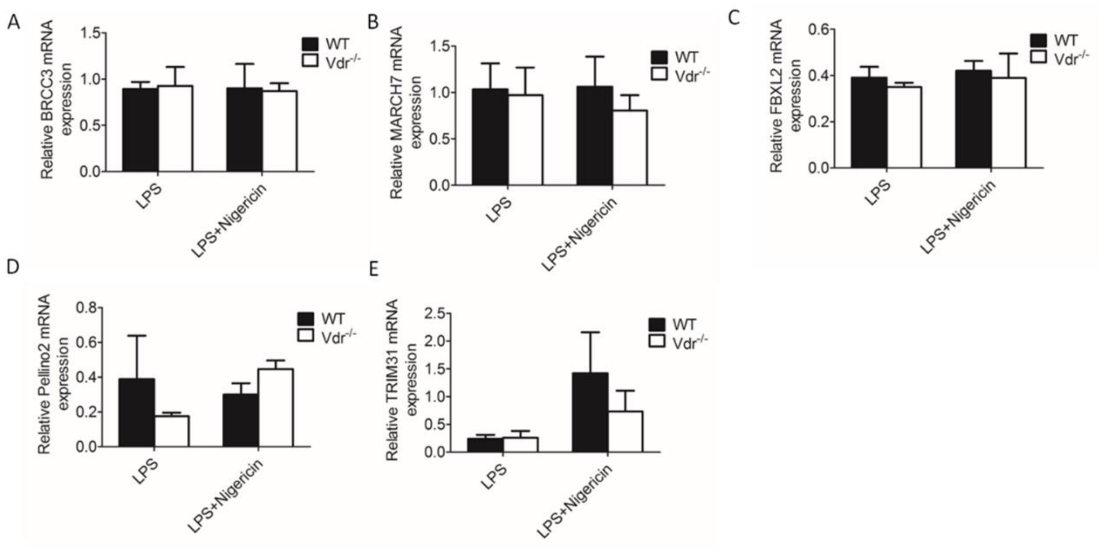

FIGURE S3.VDR mediating NLRP3 ubiquitining does not depend on transcription regulating：related to Figure 4

The mRNA expression of NLRP3 related deubiquitinase （BRCC3）(A) and ubiquitinase（MARCH7,FBXL2,Pellino2,TRIM31）(B-E) in LPS primed BMDMs (wild-type and Vdr$^{-/-}$) with or without Nigericin.

Data are representative of three independent experiments.

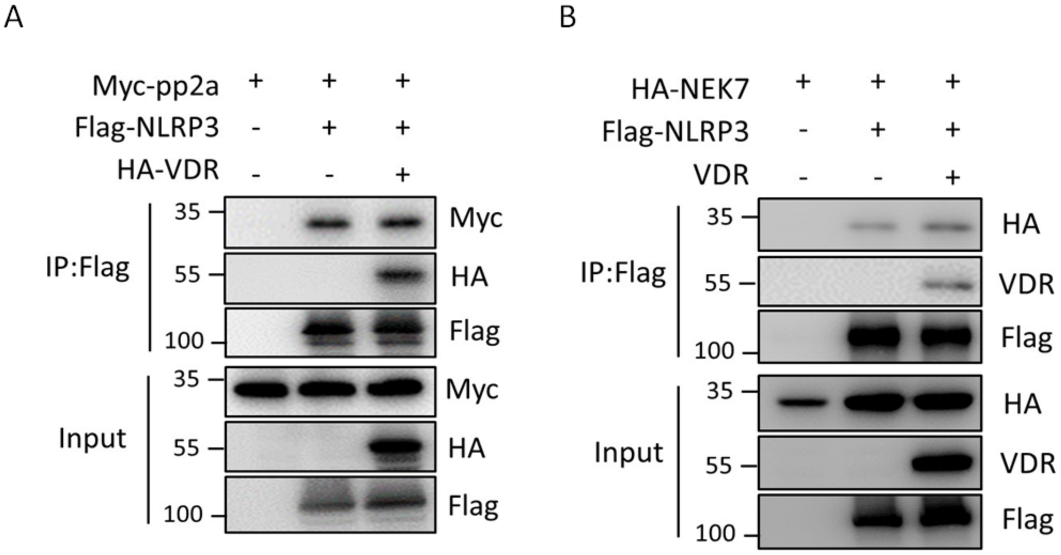

FIGURE S4. Vitamin D receptor does not interfere with the interaction between NEK7, PP2A and NLRP3: related to Figure 4

(A-B) HEK293T cells were transfected with indicated vectors. Immunoprecipitated with anti-Flag and analyzed by immunoblotting.
